# Genome scale analysis of 1-aminocyclopropane-1-carboxylate oxidase gene family in *G. barbadense* and its functions in cotton fiber development

**DOI:** 10.1038/s41598-023-30071-7

**Published:** 2023-03-10

**Authors:** Samina Yousaf, Tanzeela Rehman, Bushra Tabassum, Faheem Aftab, Uzma Qaisar

**Affiliations:** 1 Institute of Botany, University of the Punjab, Lahore, Pakistan; 2 School of Biological Sciences, University of the Punjab, Lahore, Pakistan

**Keywords:** Molecular biology, Plant sciences

## Abstract

A class of proteins, 1-aminocyclopropane-1-carboxylate oxidase (ACO), is required in the final step of production of ethylene from its immediate precursor 1-aminocyclopropane-1-carboxylic acid (ACC). Despite the crucial and regulatory role of ACO gene family in the fiber development, it has not been thoroughly analyzed and annotated in *G. barbadense* genome. In the present study, we have identified and characterized all isoforms of ACO gene family from genomes of *Gossypium arboreum*, *G. barbadense*, *G. hirsutum* and *G. raimondii*. Phylogenetic analysis classified all ACO proteins into six distinct groups on the basis of maximum likelihood. Gene locus analysis and circos plots indicated the distribution and relationship of these genes in cotton genomes. Transcriptional profiling of *ACO* isoforms in *G. arboreum*, *G. barbadense* and *G. hirsutum* fiber development exhibited the highest expression in *G. barbadense* during early fiber elongation*.* Moreover, the accumulation of ACC was found highest in developing fibers of *G. barbadense* in comparison with other cotton species. ACO expression and ACC accumulation correlated with the fiber length in cotton species. Addition of ACC to the ovule cultures of *G. barbadense* significantly increased fiber elongation while ethylene inhibitors hindered fiber elongation. These findings will be helpful in dissecting the role of ACOs in cotton fiber development and pave a way towards genetic manipulations for fiber quality improvement.

## Introduction

**Figure 1:**
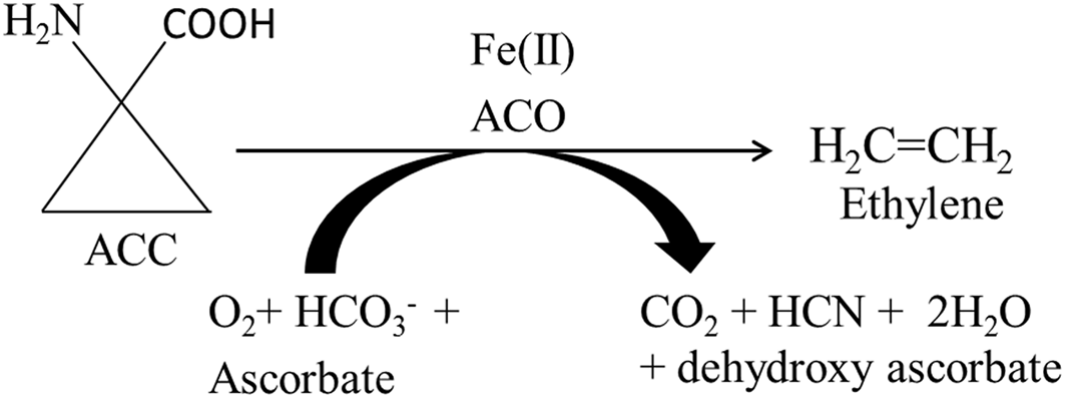
Schematic representation of ethylene biosynthesis by the activity of 1-aminocyclopropane-1-carboxylate oxidase (ACO).

Cotton fiber produced by the *Gossypium *spp. is the global source of natural fiber used in the textile industry. Cotton fiber is a single-celled trichome produced on the seed testa and the elongation of this trichome determines the length of mature fiber i.e. the most desirable characteristic for ginning industry. Although, the complete mechanism of cotton fiber elongation has not been fully understood but ethylene production is known to directly affect cotton fiber cell elongation^1^ by modulating the expression of multiple classes of genes. Ethylene serves as a signaling molecule due to its solubility in lipids, making its diffusion easier across the membranes^2,3^. The presence of endogenous ethylene precursor, 1-aminocyclopropane-1-carboxylic acid (ACC), is essential for ethylene synthesis in higher plants^4,5^. ACC is oxidized into ethylene^6^ by ACO proteins (Fig. 1). Ethylene synthesis is under the strict control of 1-aminocyclopropane-1-carboxylate oxidase (*ACO*) regulated by plant’s developmental and environmental state^7,8^. Thus the gene expression of ACO isoforms determines the rate of ethylene evolution and ACO can be the rate limiting factor for ethylene production^9,10^. Down regulation of ACO isoforms through gene silencing techniques resulted in a drastic reduction in ethylene biosynthesis^11^.

A transcription factor (MYB) has been extensively studied for its role in secondary cell wall biosynthesis^12,13^ and the presence of MYB binding sites in the promoters of *ACOs* shows the involvement of this ACO family in fiber length regulation^14^. Moreover, the upregulation of an ethylene responsive transcription factor *WRI1* and its correlation with fiber length has been reported in the developing fibers of cotton^15^. Recently, upregulation of ACO in *G. arboreum* developing fiber supports its direct involvement in fiber elongation with a positive correlation between the fiber length and the amount of ethylene production^16^. All these findings provide strong bases for studying the mechanism of action of *ACOs* in cotton fiber development. Among higher plants, the main hindrance for the analyses of *ACO* multigene family is the incomplete information of all the gene family members. The present study focuses on the identification and characterization of ACO homologues in Egyptian cotton (*G. barbadense*) and their role in fiber elongation.

## Results

### Genome wide *ACO* gene family distribution

In the present study, we conducted genome wide survey of four cotton species i.e. *G. arboreum* (A2-genome), *G. raimondii* (D5-genome), *G. hirsutum* (AD1-genome) and *G. barbadense* (AD2-genome) and identified all the ACO homologues. For this purpose, nucleotide sequences of functional *ACO1*, *ACO2*, *ACO3 *and* ACO4*^10^ were used as query sequences to retrieve all homologues in the above mentioned four cotton genomes. A total 34 unigenes were identified in which 5 belonged to *G. arboreum*, 6 to *G. raimondii*, 11 to *G. barbadense* and 12 to *G. hirsutum* genome (Table 1).

**Table 1:** Characteristics of ACO isoforms in *Gossypium* spp.

Family name	Gene name	Gene identifier	Chromosome ID	Location on chromosome	Size (Protein)	Mol. wt. (kDa)	pI (pH)
ACO1	*GaACO1*	Cotton_A_04648	chr10	19937965..19939419+	319	36.22	4.89
	*GbACO1a*	Gbar_D05G017800	D05	15336976..15339130−	319	36.19	4.76
	*GbACO1b*	Gbar_A05G017390	A05	16208845..16210132−	319	36.24	4.76
	*GhACO1a*	Gohir.D05G176900	D05	15814099..15815878−	319	36.22	4.76
	*GhACO1b*	Gohir.A05G173900	A05	17009038..17010926−	319	36.22	4.76
	*GrACO1*	Gorai.009G182300	Chr09	14025474..14027500−	319	36.18	4.89
ACO2	*GaACO2*	Cotton_A_30303	Chr08	13032853..13033998−	319	36.32	4.82
	*GbACO2a*	Gbar_A06G016410	A06	105343878..105345414+	319	36.24	4.82
	*GbACO2b*	Gbar_D06G017100	D06	54168262..54169048+	319	36.24	4.82
	*GhACO2a*	Gohir.A06G152100	A06	111491795..111493243+	319	36.24	4.82
	*GhACO2b*	Gohir.D06G158400	D06	57652944..57653411+	285	32.69	4.86
	*GrACO2*	Gorai.010G184900	Chr10	53587257..53588697	329	37.36	5.33
ACO3	*GaACO3*	Cotton_A_03276	Chr03	27810580..27811784−	317	35.96	5.08
	*GbACO3a*	Gbar_D08G005780	D08	6492710..6494232−	317	35.90	5.08
	*GbACO3b*	Gbar_A08G005560	A08	7036961..7038460−	317	35.90	5.08
	*GhACO3a*	Gohir.D08G060200	D08	6965291..6966783−	317	35.90	5.08
	*GhACO3b*	Gohir.A08G050300	A08	6763260..6764777−	317	35.97	5.09
	*GrACO3*	Gorai.004G062100	Chr04	6172557..6174159+	317	35.88	5.08
ACO4	*GaACO4*	Cotton_A_34794	Chr01	129377369..129378389−	310	35.25	5.07
	*GbACO4a*	Gbar_A07G009060	A07	13911157..13912605−	316	35.81	5.34
	*GbACO4b*	Gbar_D07G009430	D07	11224176..11225946+	313	35.63	5.01
	*GhACO4a*	Gohir.A07G084000	A07	13284835..13286217+	315	35.62	5.33
	*GhACO4b*	Gohir.D07G090100	D07	11406221..11407676+	311	35.36	5.20
	*GrACO4a*	Gorai.001G096300	Chr01	10730549..10732036	314	35.62	4.93
ACO3-like	*GbACO3*-likea	Gbar_A11G015860	A11	18341759..18343127−	310	35.28	5.47
	*GbACO3*-likeb	Gbar_D11G016650	D11	15971328..15972927−	310	35.24	5.29
	*GhACO3*-likea	Gohir.A11G152600	A11	19223648..19225116−	310	35.32	5.47
	*GhACO3*-likeb	Gohir.D11G159500	D11	16719415..16720941−	310	35.24	5.29
	*GrACO3*-like	Gorai.007G170100	Chr07	15323125..15324718−	310	35.24	5.14
ACO4-like	*GaACO4*-like	Cotton_A_18687	Chr01	29392016..29393132+	282	32.15	7.01
	*GbACO4*-like	Gbar_D07G020460	D07	91687441..91689105+	311	35.28	6.68
	*GhACO4*-likea	Gohir.D07G194300	D07	49345636..49346959+	311	35.33	6.68
	*GhACO4*-likeb	Gohir.A07G187400	A07	83662574..83663898+	311	35.30	6.68
	*GrACO4*-like	Gorai.001G217400	Chr01	43665158..43666523+	311	35.33	6.68

The genes of *ACO* isoforms in *G. arboreum* were located on chromosome 01, 03, 08 and 10. From these *GaACO1*, *GaACO2* and *GaACO3* were located on chromosome 10, 08 and 03 respectively. On the other hand, both *GaACO4* and *GaACO4*-like were located on chromosome 01 (Table 1).

*G. raimondii* also harbored a uniform distribution of 6 *GrACO* gene members on 05 chromosomes (01, 04, 07, 09 and 10). Very similar to *G. arboreum* (A2), both *GrACO4* and GrACO4-like were located on the same chromosome (01) while *GrACO1*, *GrACO2*, *GrACO3* and *GrACO3*-like are located on chromosomes 9,10, 4 and 7 respectively (Table 1). All these uniform distribution patterns and variations provide us the clues for the evolutionary relations of the ACOs in different cotton genomes.

Similarly, among all the retrieved sequences, 11 were from *G. barbadense* tetraploid cotton (AD2). The *GbACO* genes were uniformly distributed on both A and D sub-genomes of five chromosomes (chromosome 5, 6, 7, 8 and 11). Both A and D homologues of chromosome 05 accommodate one copy of *GbACO1* each. Similarly, GbACO2 isoforms are located on chromosome A06 and D06 homologues each, while *GbACO3* homologues are located on A08 and D08. Both members of *GbACO4* and one gene of *GbACO4*-like were located on homologues of chromosome 07 (Table 1). Both homologues of *GbACO3*-like were located on A11 and D11 sub genomes.

A total of 12 *GhACOs* were identified in the *G. hirsutum* tetraploid genome (AD1) which were uniformly distributed on both A and D genomes of this allopolyploid species on chromosome 05, 06, 07, 08 and 11 (Table 1). Distribution of *ACO* isoforms in *G. hirsutum* (AD1) genome matched largely with *G. barbadense* (AD2) except for *GhACO4*-like members. *G. hirsutum* contained 2 *GhACO4*-like members, one located on each A and D sub-genome of chromosome 07 while *G. barbadense* D genome harbored *GbACO4*-like but A genome missed that (Table 1).

### Gene structure analysis for *ACO* isoforms

**Figure 2:**
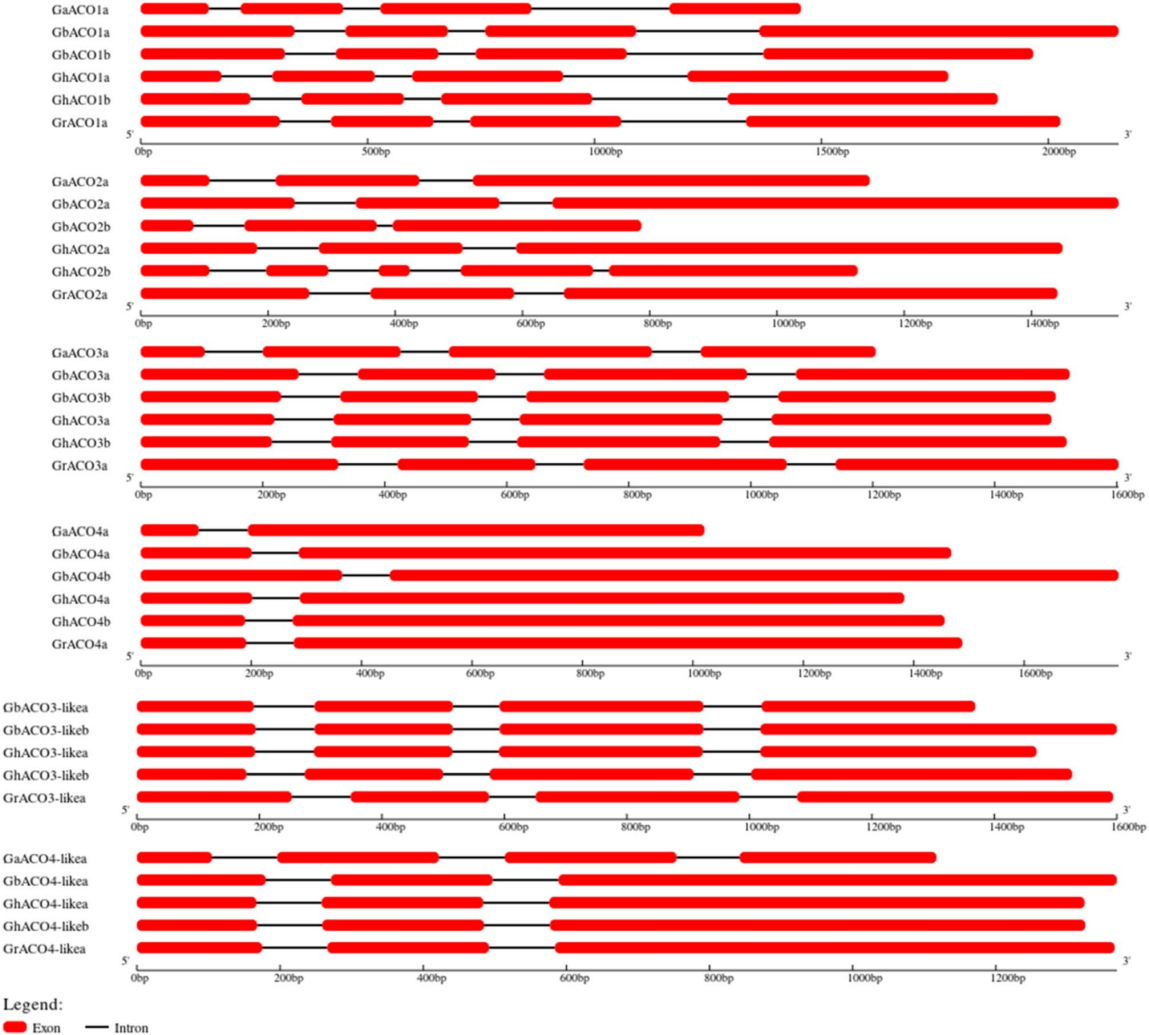
Gene structure of *ACO* isoforms showing intronic and exonic regions.

The intron–exon boundaries of all the *ACOs* were identified and gene structures were constructed (Fig. 2). All members of *ACO1* gene subfamily contained three introns and four exons which differ widely in their lengths (Fig. 2). The six members of *ACO2* subfamily showed much variation in coding region lengths as well as in the length of introns and exons. In *G. hirsutum*, the *ACO2* isoform of D genome (*GhACO2b*) contains 5 exons and 4 introns, while the rest of the members of this gene subfamily contain three exons and two introns. Presence of 4 exons and 3 introns distinguishes *ACO3* and *ACO3*-like subfamilies from *ACO4* and *ACO4*-like subfamilies, although variations in gene lengths are there (Fig. 2). In *ACO4* and *ACO4*-like subfamily, all gene members have two and three exons respectively, except *GaACO4-like* where the gene exists with four exons and three introns.

### Conserved regions in ACO isoforms of cotton species

**Figure 3:**
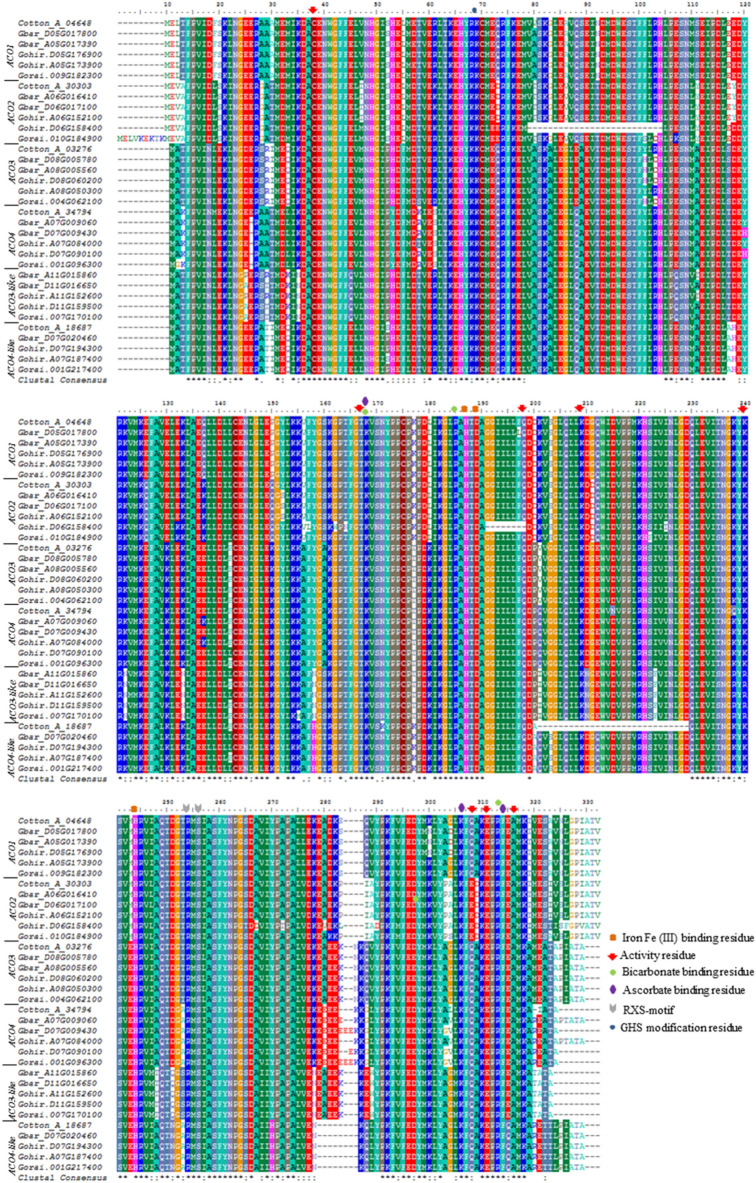
Residue analysis of ACO isoforms in *Gossypium* spp. (*G. arboreum*, *G. raimondii*, *G. hirsutum* and *G. barbadense*). Marked legends represent the important residues.

Iron (Fe II) is a cofactor required for the enzymatic activity of ACOs. Scanning of ACO proteins on Prosite database of protein domains (http://www.prosite.expasy.org) lead to the identification of Fe2OG dioxygenase domain at position 155–254 in ACO1 family of *G. barbadense* (GbACO1). This domain was found conserved among all isoforms of ACOs indicating its functional activity. In this domain triad His177 (H177), Asp 179 (D179) and His234 (H234) constitute an iron binding region^17^. We performed the residue analysis of ACOs from *G. barbadense* along with three other cotton species through multiple sequence alignment (Fig. 3) and confirmed the presence of highly conserved iron binding residues at position (H178, D180 and H235) in ACO1 and ACO2 with the exception of Gohir.D06G158400 and Gorai.010G184900 where iron binding triad is located at position H152, D154 and H201 and H187, D189 and H244 respectively. In all other ACO classes iron binding site is located at H177, D179 and H234 (Fig. 3).

Bicarbonate and ascorbate serve as activators for ACO to catalyze the oxidation of ACC to ethylene in plant species^17–19^. The residues involved in bicarbonate binding are Lysine159 (K159), Arg176 (R176) and Arg300 (R300). We found bicarbonate binding residues at position K159, R176 and R300 in *G. barbadense.* Ascorbate provides binding site for oxygen in the oxidation of ACC to ethylene^19^ and reported the specific sites for ascorbate binding in this family. In the aligned sequences of cotton, these conserved sites were identified at K159, K293 and F301.

Various other residues i.e. C29, T158, K159, R176, Q189, K200, K231, R245, S247, K293, E295, E298, R300, F301 and E302 have been described earlier in *Malus domestica*^19^ as essential residues for the activity of ACOs. Most of these residues (C29, T158, K159, R176, R245, S247, K293, E298, R300 and F301) were found conserved in all ACO isoforms. Although two residues i.e. Q189 and K200 were also found conserved but were absent in Gohir.D06G158400 and Cotton_A_18687 respectively. Interestingly, K231 was found absent in ACO3-like subfamily and has been replaced by R231, although conserved in all other ACO subfamilies. On the other hand, important activity residue E295 was found only in ACO2 subfamily while in all other subfamilies, it has been replaced by the residue Q. Similarly, E302 was found absent in ACO4-like subfamily and replaced by Q.

Another motif i.e. RXS motif (constituted by the residues R244 and S246 separated by X i.e. any amino acid) is involved in substrate binding of ACC to ACO^19^. We found conserved RMS at position 245–247 in *G. barbadense* and confirmed its conservation throughout the cotton ACO gene family. Glutathione (GSH), γ-glutamyl-cysteinyl-glycine, is a small cytosolic molecule which serves as a strong non-enzymatic antioxidant^20^ and modulates the mRNA stability of ACO by S-glutathionylation at Cys63 residue^21^. We found this residue conserved throughout the ACO family at position C61 (Fig. 3).

### Classification and phylogenetic analyses of ACO isoforms from *Gossypium* species

**Figure 4:**
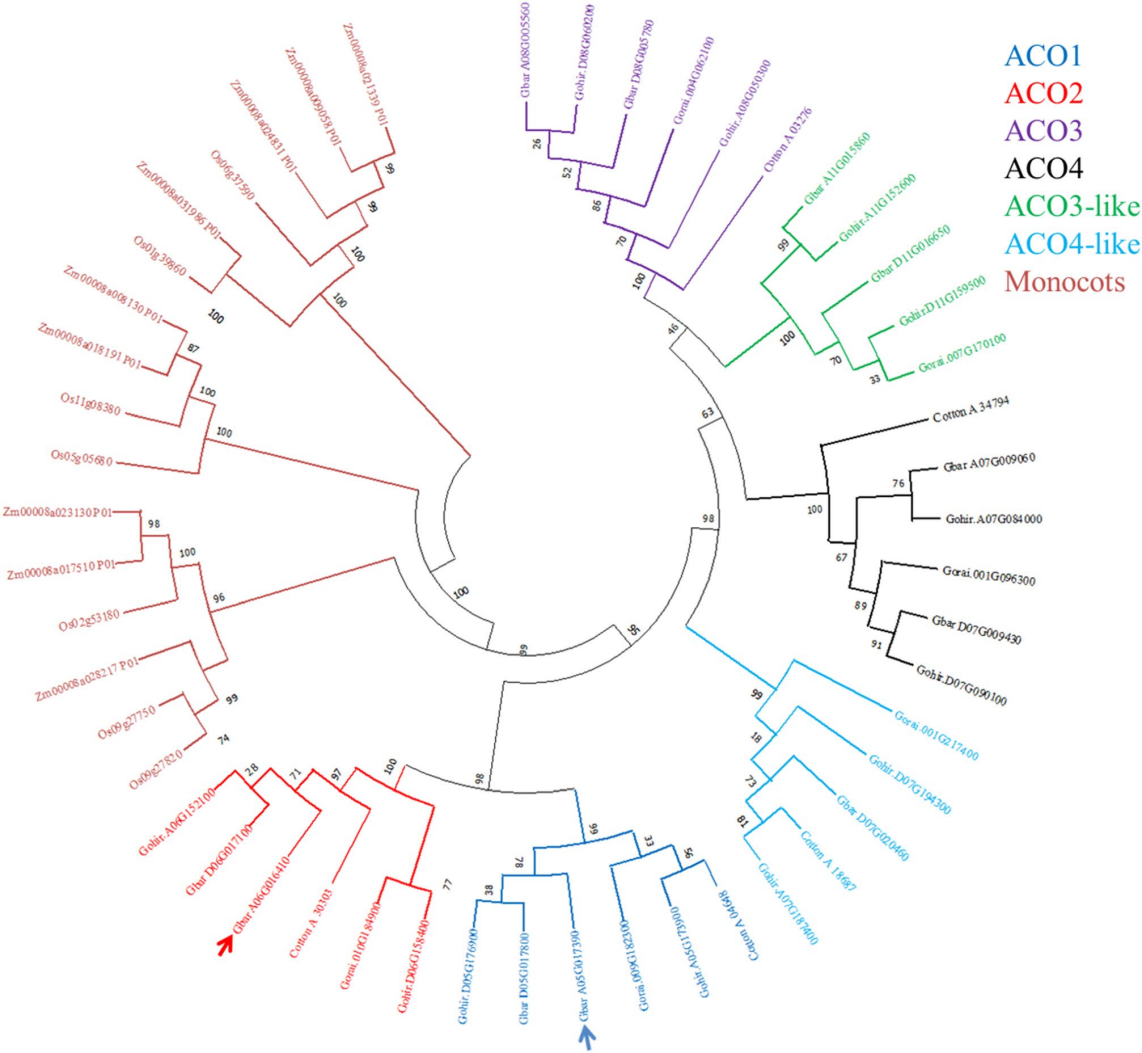
Classification of ACO isoforms in *Gossypium* spp. (*G. arboreum*, *G. raimondii*, *G. hirsutum* and *G. barbadense*) and monocotyledonous species (*O. sativa* and *Z. mays*). Arrow heads indicate the homologous sequences of isolated genes. The number at each node indicates the percent bootstrap value.

ACO1 (Gbar_A05G017390) and ACO2 (Gbar_A06G016410) coding regions were isolated from *G. barbadense* var Bar 14/5 cDNA library and sequenced. The amino acid sequences of *G. barbadense* ACO isoforms were obtained and aligned with the ACOs from other cotton species to find the evolutionary relationship. Phylogenetic tree was constructed through maximum likelihood method with 1000 bootstrap value (Fig. 4). The peptide sequences of GbACO1 and GbACO2 from *G. barbadense* were also included in this analysis to find their phylogenetic relevance with ACO homologues. The isolated GbACO1 and GbACO2 exhibited 99 and 99.5% homology with GbarA05G017390 and GbarA06G016410, respectively. Phylogenetic analysis distributed the ACO gene family into four ACO1, ACO2, ACO3 and ACO4 subfamilies. In addition to these, two ACO-like (ACO3-like and ACO4-like) subfamilies were also identified (Fig. 4).

Four ACO subfamilies (ACO1, ACO2, ACO3 and ACO4) were constituted by six gene members i.e. one from *G. arboreum*, 2 from *G. barbadense*, 2 from *G. hirsutum and* one from *G. raimondii*. ACO3-like subfamily comprised of five gene members having 2, 2 and one homologues from *G. barbadense*, *G. hirsutum* and *G. raimondii*, respectively (Fig. 4). ACO4-like subfamily also comprised of five homologues. This subfamily constituted one member from *G. arboreum*, one from *G. barbadense*, two from *G. hirsutum* and one from *G. raimondii*.

### Genomic divergence of ACO families in *Gossypium* spp.

**Figure 5:**
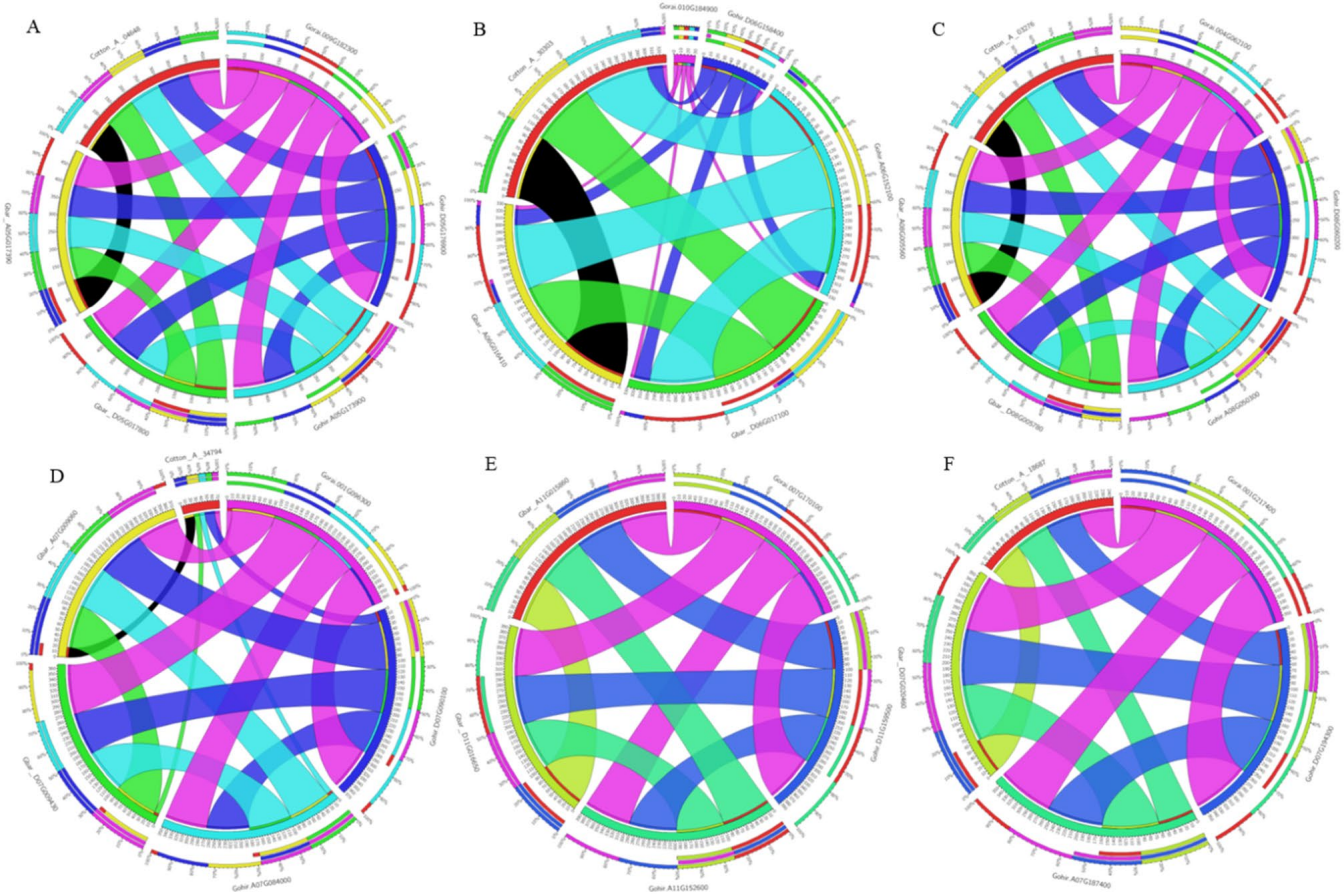
Sub-genomic characterization of *ACO* (1-aminocyclopropane-1-carboxylate oxidase) subfamilies (**A**) ACO1, (**B**) ACO2, (**C**) ACO3, (**D**) ACO4, (**E**) ACO3-like, (**F**) ACO4-like. The thickness of the bands represents the percent homology while the colors of the band represent the cotton species from which they originate.

We constructed the circos figures to observe the trends and correlations among the members of ACO subclasses in A and D subgenomes (Fig. 5). Individually, all members of ACO1 subclass shared high homology (98.4–99.7%) with other members of ACO1 family, thus showing lesser diversity within this sub-class (Fig. 5A). Relative sequence complexity and diversity was observed while comparing the gene members of ACO2 sub-class as shown in Fig. 5B. In this subclass, *G. arboreum* (Cotton_A_30303), both A and D genome of *G. barbadense* (Gbar_A06G016410 and Gbar_D06G017100) and A genome of *G. hirsutum* (Gohir.A06G152100) showed maximum and approximately equal (more than 99%) homology with each other. Moreover, D genome of *G. hirsutum* (Gohir.D06G158400) showed approximately 24% homology with others except *G. raimondii* (Gorai.010G184900) where only 5.4% homology could be observed. Thus, ACO2 from *G. raimondii* (Gorai.010G184900) showed maximum diversity in comparison to other gene members (Fig. 5B).

In case of sub-class ACO3, the homology among its members was 98.7–100% indicating common ancestors (Fig. 5C). While ACO4 subclass shows diverse homology patterns among its members i.e. 10.2–99% (Fig. 5D). ACO4 of *G. raimondii* shares more than 84% homology with the A and D sub-genomes of both allotetraploids i.e. *G. hirsutum* and *G. barbadense* (Fig. 5D). ACO4 of *G. arboreum* (Cotton_A_34794) shows only 10–20% homology with other members of this subclass. ACO3-like sub-class indicates less variability similar to ACO3 subclass (Fig. 5E). Within this sub-class, more than 97% homology was observed when all members were compared to each other. ACO4-like isoform from *G. raimondii* shows more than 98% homology with other members of this sub-class (Fig. 5F) indicating common ancestors while ACO4-like from *G. arboreum* (Cotton_A_18687) shows 61–62% homology with other members of this family.

### Functional modulation of *ACOs* during cotton fiber development

**Figure 6:**
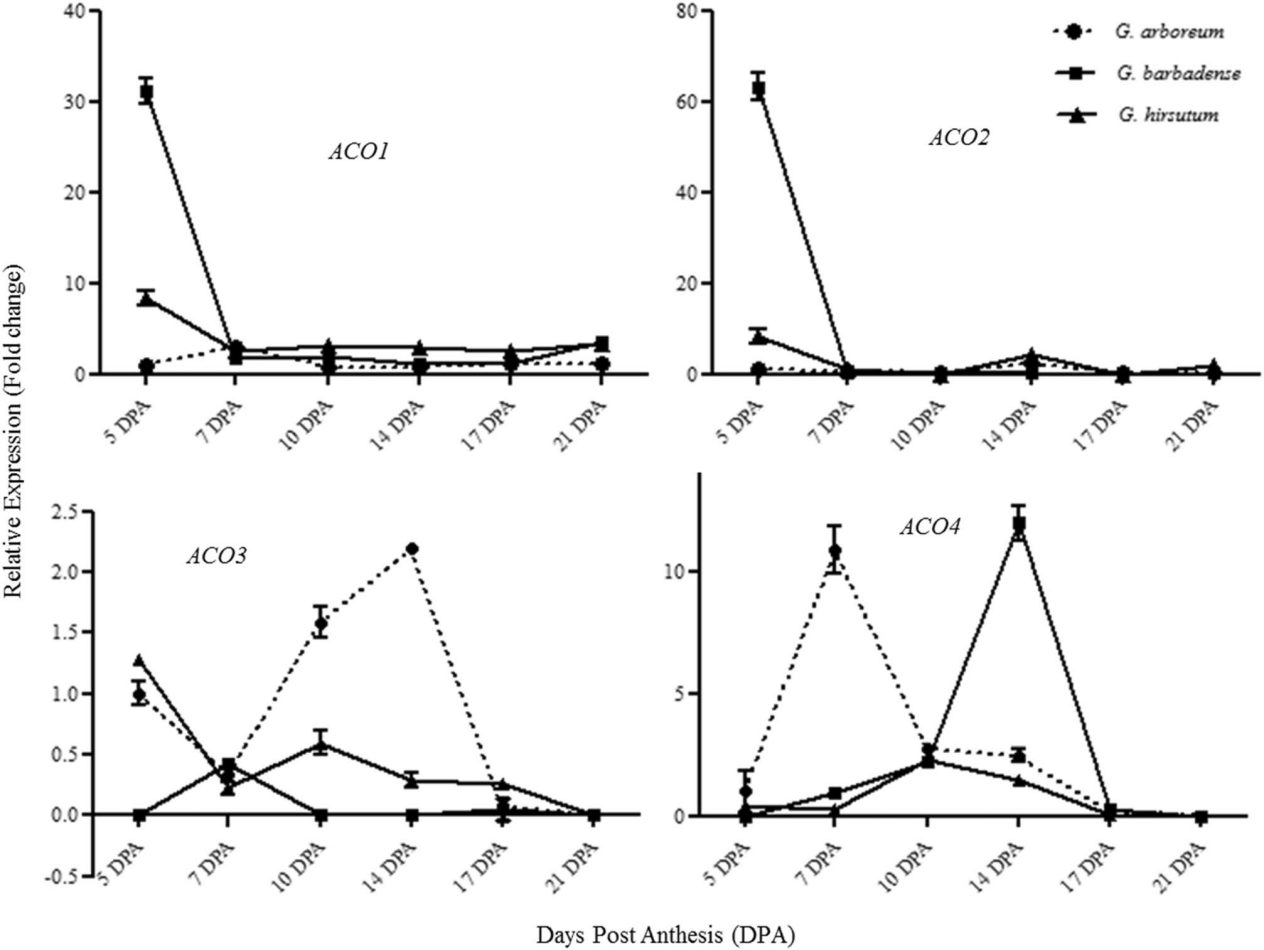
Transcription abundance of *ACO* isoforms in developing fibers of *Gossypium* spp.

Cotton fiber development is a very important process culminating in the formation of natural fiber required in the textile industry. Transcript abundance of *ACO* isoforms was measured through realtime RT-PCR in different cotton species at 5, 7, 10, 14, 17 and 21 DPA developing fibers. Temporal expression of *ACO1* and *ACO2* gene was found highest in *G. barbadense* in comparison with other cotton species during early fiber elongation stage (5 DPA). The *ACO1* expression was 31 fold higher at 5 DPA in the fibers of *G. barbadense* and 8.4 fold in *G. hirsutum* in comparison with *G. arboreum* (Fig. 6). The *ACO2* expression was found 63 fold and 8 fold higher in *G. barbadense* and *G. hirsutum* in comparison with *G. arboreum* respectively. In the later stages of fiber development, there was a drastic decrease in *ACO1* and *ACO2* expression in both species. In *G. arboreum*, the relative expression of *ACO1* and *ACO2* remained almost at bottom line throughout the fiber elongation process. Similar expression pattern of both genes indicate functional redundancy of these genes.

*ACO3* showed higher transcriptional abundance at 5 DPA in *G. arboreum* in comparison with other species. This expression decreased at 7 DPA but showed a gradual rise and reached at its peak at 14 DPA and again reached at basal line at 17 DPA (Fig. 6C). Almost the same but low pattern of transcriptional expression was observed in *G. hirsutum*. On the other hand, in *G. barbadense*, the transcriptional expression remained highest at 7 DPA which decreased to basal line throughout its later fiber developmental stages. In case of *ACO4*, at 7 DPA the transcriptional abundance was found highest in *G. arboreum* while of the other two species remained almost at bottom line. At 10 DPA, it was again almost at the same level in the three cotton species. Interestingly, a rise in transcriptional expression was observed only in *G. barbadense* at 14 DPA. At 17 DPA, the transcriptional expression was almost at basal line in all species (Fig. 6D).

### 1-Aminocyclopropane-1-carboxylic acid (ACC) content during cotton fiber development

**Figure 7:**
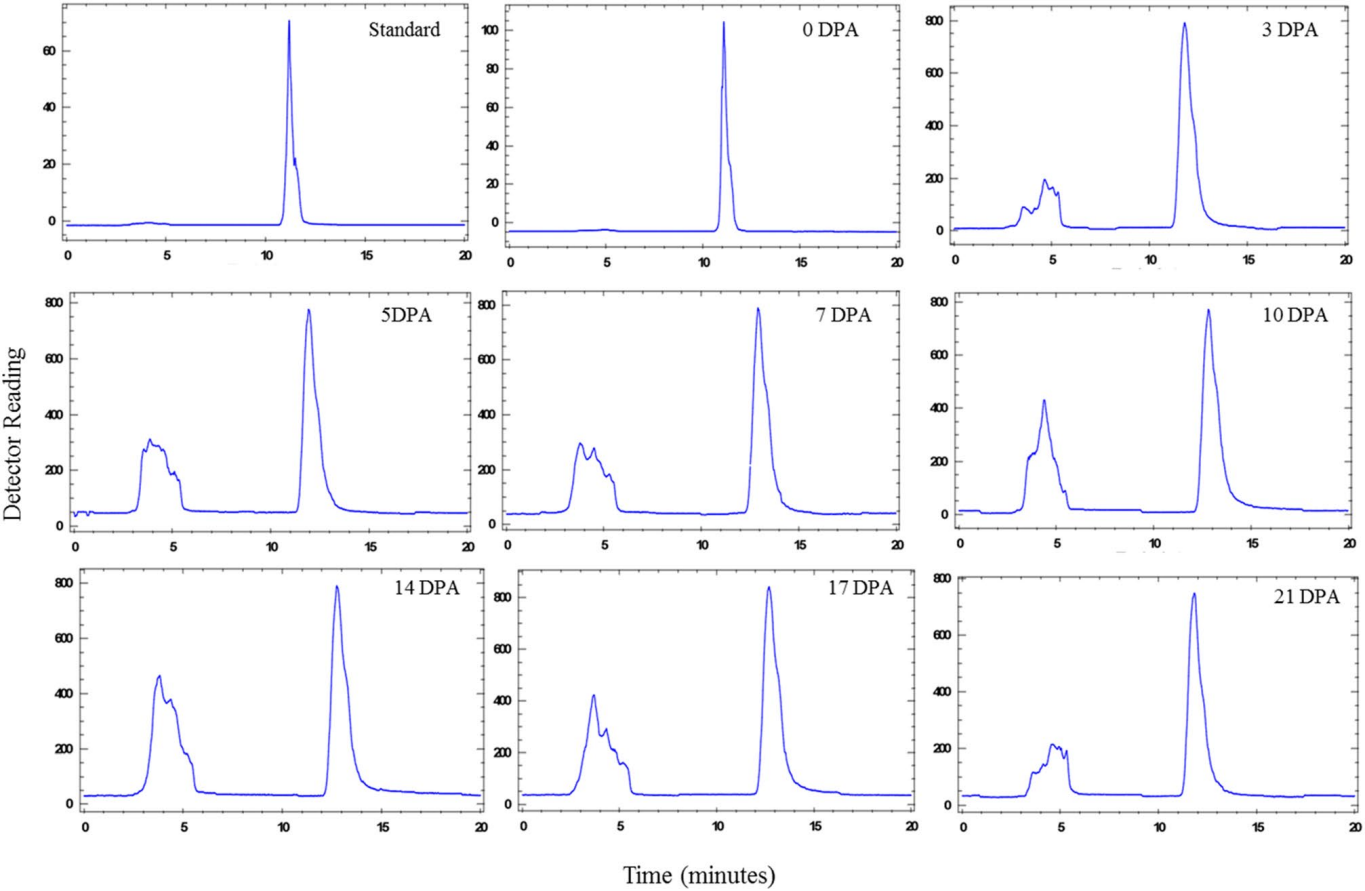
1-Aminocyclopropane-1-carboxylic acid (ACC) chromatogram obtained through high performance liquid chromatography (HPLC) in the developing fibers of *G. barbadense.*

**Figure 8:**
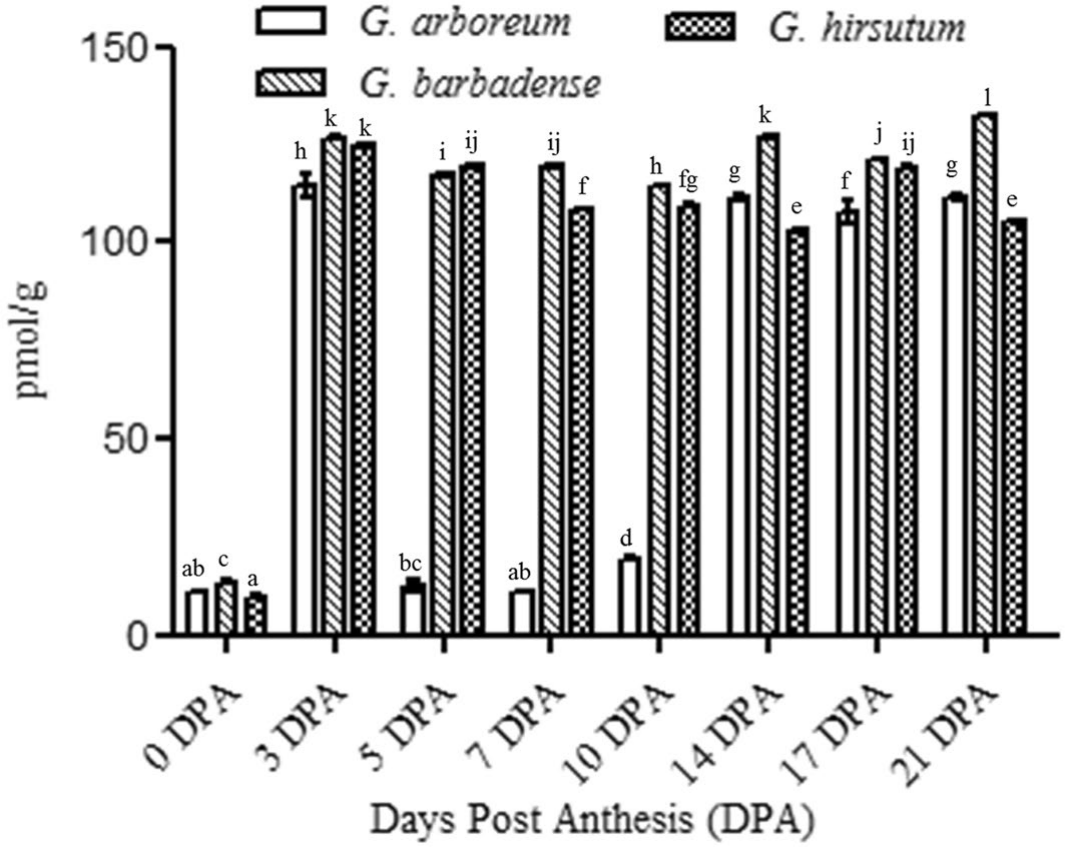
Endogenous 1-aminocyclopropane-1-carboxylic acid (ACC) content (pmol/g of tissue) in developing fibers through high performance liquid chromatography (HPLC).

**Figure 9:**
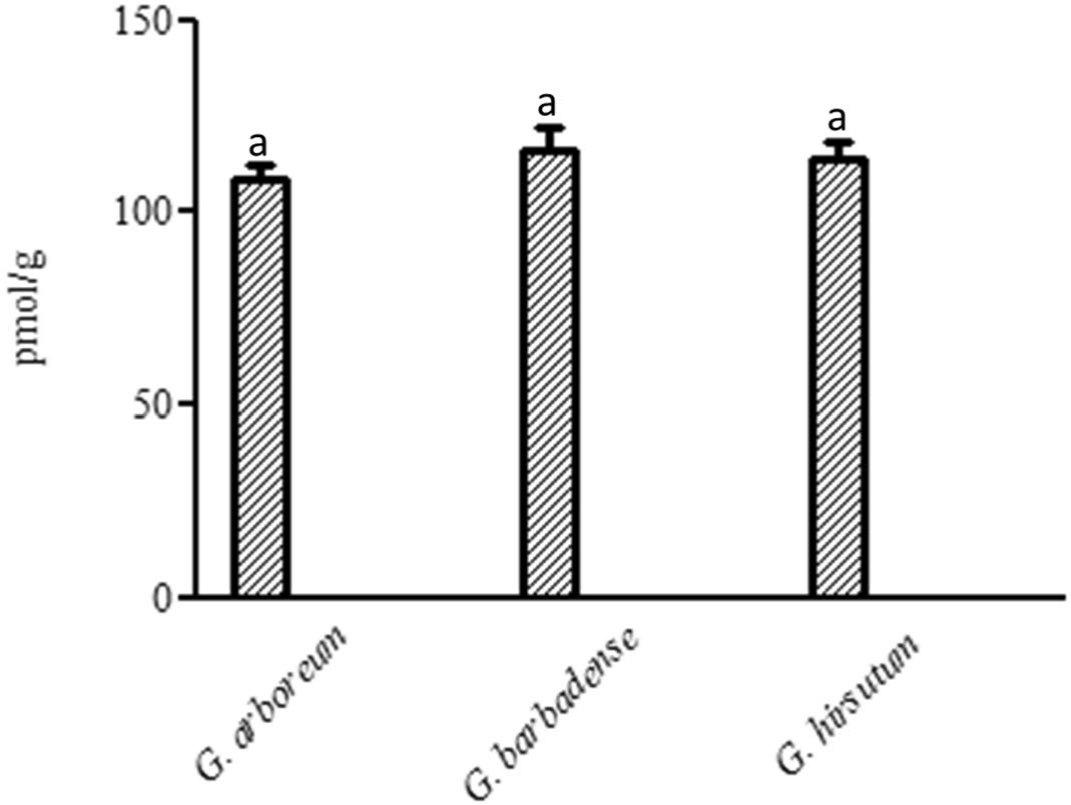
Endogenous 1-aminocyclopropane-1-carboxylic acid (ACC) content in the leaf tissue of cotton through high performance liquid chromatography (HPLC).

As ACC is the substrate of ACOs for ethylene production, so we wanted to quantify the endogenous ACC pool in cotton fibers of *G. barbadense* in comparison with other cotton varieties. We determined ACC content in cotton fibers at different developmental stages through high performance liquid chromatography (Fig. 7). The endogenous ACC level was found highest in *G. barbadense* throughout the fiber developmental stages as compared to *G. arboreum* (Fig. 8). At 0DPA, the endogenous ACC concentration was approximately 11, 13 and 10 pmol/g of fiber in *G. arboreum*, *G. barbadense* and *G. hirsutum* respectively. During fiber elongation stage (3 DPA), a sudden rise in ACC concentration was observed in the three cotton species. In *G. barbadense*, at 3 DPA it was approximately 126 pmol per gram of the tissue while at 21 DPA it reached to its peak i.e. 132 pmol/g of fiber with the slight fluctuations during intermediate fiber developmental phases (Fig. 8). *G. arboreum* showed a drastic decline in ACC concentration from 114 pmol to only 12 pmol/g fiber from 3 to 5 DPA. Again at 14 DPA it restored its endogenous concentration up to 112 pmol/g fiber which remained almost static during further fiber developmental stages. Contrary to *G. barbadense* which had highest ACC concentration at 21 DPA fibers, *G. hirsutum* showed highest ACC concentration at initial fiber developmental phase. At 3 DPA it reached up to 124 pmol/g of fiber. After a small decline during further fiber developmental stages it again reached up to 119 pmol/g fiber at 17 DPA. Moreover, the endogenous ACC concentration was measured in the vegetative leaves of cotton species. It was 108.137, 116.344 and 111.95 pmol/g of leaf tissue in *G. arboreum*, *G. barbadense* and *G. hirsutum* respectively (Fig. 9).

### Effect of ACC, AgNO_3_ and CoCl_2_ on fiber length

**Figure 10:**
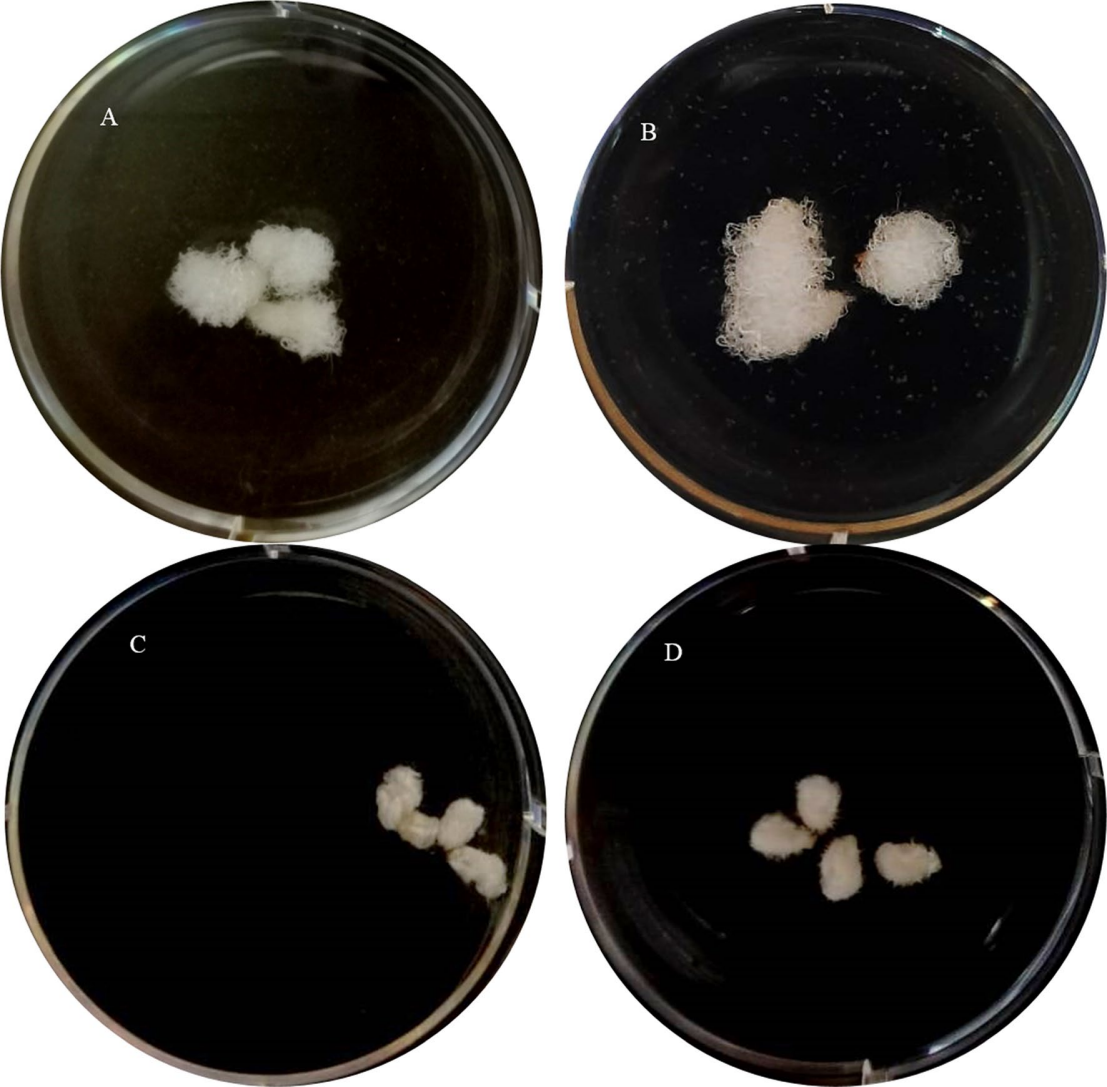
Ovule cultures of *G. barbadense* in modified BT medium^22^ at 21 DPA (**A**) control BT without any supplement (**B**) medium supplemented with 10 µM ACC (**C**) 40 µM of CoCl_2_ (**D**) 30 µM of AgNO_3_.

Ovules of *G. barbadense* at 0DPA were cultured in BT medium^22^ and fiber growth was documented at 21 DPA. When cultures were supplemented with ACO substrate (ACC) for the production of ethylene, relatively longer fibers (23 mm) were observed as compared to un-supplemented control where fiber length was 18 mm (Fig. 10A,B). When cultures were supplemented with previously reported^23^ ethylene inhibitors (AgNO_3_ and CoCl_2_), the fiber growth was retarded (Fig. 10).

## Discussion

Ethylene is an important growth factor participating in various physiological and developmental processes in higher plants under normal as well as stress conditions^24–26^. In higher plants, 1-aminocyclopropane 1-carboxylic acid (ACC) is the immediate ethylene precursor converting to ethylene and this bioconversion is under the control of ACC-oxidase enzymes. Molecular oxygen and ascorbate serve as the co-substrates during this biochemical conversion^11^. In *G. hirsutum*, four ACO isoforms namely *ACO1*, *ACO2*, *ACO3* and *ACO4* have been identified^10^. In a recent report, 20 ACOs have been identified in *G. hirsutum*^1^. In other plants, numerous members of this family have been described and their classification is thus a complex phenomenon.

*ACOs* and ethylene production play vital role in the fiber elongation (Fig. 10) and *G. barbadense* is the longest and finest fiber producing species as shown in our published data^15^. So it’s very important to thoroughly investigate ACOs family in *G. barbadense* and compare it with the other tetraploid and diploid species of cotton. In the present work, two tetraploid cotton species, i.e., *G. barbadense* and *G. hirsutum* and two diploid species belonging to ancestral genome A (*G. arboreum*) and D (*G. raimondii*) were used to study the ACO gene families. ACO isoforms were identified in the genomes of tetraploid and diploid cotton species and molecular characterization was performed. Phylogenetic analysis grouped all these isoforms in four classes of ACOs and two ACO-like classes (Fig. 4). The gene structure analyses also revealed the structural variations and gene architecture among these classes (Fig. 2).

Gene expression of *ACOs* determine ethylene production and silencing of *ACO* resulted in a drastic reduction in ethylene biosynthesis^11^. In order to access the amount of ethylene production at different stages of fiber development in diploid and tetraploid varieties of cotton, we determined the expression of ACOs as well as concentration of ACC in the developing fibers. Among the cotton species under study, *G. barbadense* revealed the highest level of *ACO1,* and *ACO2* transcript accumulation during early fiber elongation phase and upregulation of *ACO4* at later developmental stages (Fig. 6). Upregulation of *ACO* genes has been described to promote cell wall loosening and changes in cytoskeletal arrangement to facilitate fiber elongation^27^. *ACO1* and *ACO2* transcription levels exhibited here correlate with the lengths of mature fibers. *G. barbadense* produces longest cotton fibers followed by *G. hirsutum* and *G. arboreum* respectively^15^. *G. hirsutum* which is the second longest fiber producing species, reveals the activation of *ACO3* along with *ACO2* and *ACO1* during early fiber elongation stages while *G. arboreum* displays activation of *ACO3* and *ACO4* during fiber elongation (Fig. 6).

Endogenous ACC pool which serves as a substrate for ethylene production has been described as limiting factor in ethylene production^4,5^. We investigated this important component and found that ACC pool was biggest in the vegetative leaf tissue as well as in the developing fibers of *G. barbadense* in comparison with *G. hirsutum* and *G. arboreum* (Figs. 8, 9). We also studied the effect of ethylene production on fiber length in *G. barbadense* by growing ovules in medium supplemented with ethylene precursor or ethylene inhibitors. We found that the ethylene precursor which is the substrate of ACOs enhanced while ethylene inhibitors reduced fiber length (Fig. 10). In our previous studies, the expression of *wrinked-1*, an ethylene responsive transcription factor showed highest expression in fibers of *G. barbadense* as compared to *G. hirsutum* and *G. arboreum*^15^. Transformation studies of *Gbwri1* (EREB) in *Arabidopsis thaliana* have also demonstrated its role in fiber development^28^. The highest expression of this ethylene responsive transcription factor might also play a supportive tool in exploring the role of ethylene in fiber development. From our findings, in this study, it is quite obvious that ethylene biosynthesis is a complex phenomenon and can be affected by various factors like the presence of its precursor, the fiber developmental phase, etc. Furthermore, the promoter analyses and further investigations of ACO classes might be helpful in exploring and understanding the role of ethylene in cotton fiber development.

## Conclusion

The relative expression of *ACOs* in cotton fibers at different stages of fiber elongation in *G. arboreum, G. barbadense* and *G. hirsutum* will further support the role of ACC oxidases in fiber cell elongation. The correlation between the endogenous ACC pool in the leaves and at different fiber developmental stages of the three *Gossypium* species also support the possible role of ethylene in cotton fiber elongation. Moreover, addition of ACO substrate enhances fiber length of *G. barbadense* in ovule cultures while ethylene inhibitors hinder fiber growth.

## Materials and methods

### Plant growth conditions and sampling

The seeds of *G. arboreum* (cv. FDH786), *G. barbadense* (cv. bar14/5) and *G. hirsutum* (cv. MNH886) were procured from Central Cotton Research Institute (CCRI), Multan, Pakistan, as a gift. FDH786 is a Pakistani origin cultivated variety of *G. arboreum*^15,29,30^ developed in University of Agriculture Faisalabad and can be searched on cottongen too. Bar 14/5 is Egypt origin germplasm present in the germplasm pool of CCRI, Multan while MNH886 of *G. hirsutum* is the commonly cultivated variety of Pakistan developed in 2012 (https://aari.punjab.gov.pk/cri_multan_achievements). Mature fibers of all the above three varieties were tested for fiber quality traits and published in a separated study^15^. During the cotton growing season (March–September 2019), seeds were sown in the field of School of Biological Sciences, University of the Punjab, Lahore under natural conditions following the standard protocols and good agricultural practices. Cotton flowers were tagged on the first day of opening of the flower and bolls at different developmental stages were harvested on ice and dissected to isolate the developing fibers which were immediately immersed in liquid nitrogen and stored in − 80 °C freezer^15^. Bolls were collected at multiple developmental stages i.e. 5, 7, 10, 14, 17 and 21 days’ post anthesis (DPA) from all cotton species under study. All methods and procedures were carried out in accordance with the relevant guidelines. *G. raimondii* was not included in the fiber related experiments due to its inability to produce spin-able fibers.

### Identification and in-silico analysis of ACOs

Previously reported nucleotide sequences of *ACO1* (DQ116442), *ACO2* (DQ116443), *ACO3* (DQ116444) and *ACO4* (DQ122175)^10^ were used to search for the homologues in *G. arboreum* (A-genome)*, G. raimondii* (D5-genome), *G. barbadense* (AD2-genome) and *G. hirsutum* (AD1-genome) sequences (CottonGen database) through nucleotide basic local alignment search tool (BLASTN). Splice/Sequence variants were combined to form unigenes. Amino acid sequences of the homologues were retrieved and aligned through ClustalW algorithm while phylogenetic tree was constructed using Maximum Likelihood method with 100 bootstrap values using pairwise deletion option with Poisson substitution model on MEGA version X^31^. An online resource, DNA Pattern Find was used to predict the molecular weights and Isoelectric points (pI). Moreover, Gene Structure Display Server (GSDS) was used to generate gene structure for all *ACO* homologues^32^. The chromosomal locations of all the *ACO* isoforms were analyzed in the Jbrowser portal of Cottogen database. Circos online tool was used to find the evolutionary relationship between diploid and tetraploid species of cotton (http://circos.ca).

### Transcript profiling

From the developing fiber samples, RNA was isolated through modified hot borate method^33^. Complimentary DNA (cDNA) was synthesized from the isolated RNA samples using Invitrogen SuperScript™ II RT First Strand cDNA Synthesis kit using random hexamer primers. Forward and reverse primers (18–30 bp) were designed (Table 2) to study gene expression in the *Gossypium* species through online PRIMER3 software^34^. ACO1 and ACO2 primers were designed with the objective to amplify only family specific products. While ACO3 primers possessed the ability to amplify *ACO3* and *ACO3-like* products and similarly ACO4 primers amplified both *ACO4* and *ACO4-like* products. After primer optimization, quantitative expression was measured using Sybr green master mix in PikoReal thermocycler (Thermo Scientific, Waltham, MA, USA). Elongation Factor 1 (EF1) was used as the endogenous control to normalize *ACOs* expression during development. At least three biological and three technical replicates were used for every sample^15,35^.

**Table 2:** Primer sequences used in this study.

No.	Primer ID	5ʹ…3ʹ sequence	Product size (bp)	Tm (°C)
Gene expression analysis				
01	ACO1-F	gctgacctcaagttccaagc	211	60.00
02	ACO1-R	tgcaggcactctctaattcca		60.99
03	ACO2-F	acaaccctggaagtgatgct	216	59.58
04	ACO2-R	aatgggtggttcaaacagttg		59.74
05	ACO3-F	aggtttgaagccatgaaagc	156	59.32
06	ACO3-R	ttcaacagtgcaaacaacacac		59.71
07	ACO4-F	ttgctggagaaagaagaagaaga	210	59.77
08	ACO4-R	aacgataagatttccaggttgg		59.37
Gene isolation				
09	GbACO1 -F	ggacctttttagttttgcagtatctat	1949	58.80
10	GbACO1-R	aaaaacagaatcttaaggacaattttt		58.40
11	GbACO2 -F	cccctcactttgtgcctataaa	1500	60.36
12	GbACO2-R	aatgggggaattgcagtagttag		60.58

### Isolation and sequencing of *ACO1* and *ACO2* from *G. barbadense*

For isolating *ACO* genes, cDNA was synthesized from RNA isolated from the developing fibers (10 DPA) of *G. barbadense* using Oligo (dT) primers. From the cDNA library, full length sequences of *ACO1* (*GbACO1*) and *ACO2* (*GbACO2*) were amplified using gene specific primers (Table 2). The Amplified *GbACO1* and *GbACO2* were then cloned in pJET cloning vector using CloneJET PCR Cloning Kit (Thermo Scientific, MA, USA). The constructs were sequenced using insert flanking universal primers^36^.

### ACC content estimation through high performance liquid chromatography (HPLC)

ACC endogenous pool was measured in developing fibers (5, 7, 10, 14, 17 and 21 DPA) and the whole gynoecium at 0 and 3 DPA, of the three cotton species under study. For this quantification, we used the modified method^37^ by preparing ACC derivative with *o*-phthaldialdehyde (OPT). Briefly, for ACC extraction, fibers were ground to fine powder and 5 ml of ice-chilled 70% ethanol were added to it. The mixture was homogenized thoroughly and was then centrifuged at 10,000 rpm for 15 min at 4 °C. The supernatant was filtered through the 0.2 µm micro-filter. The working samples were prepared by dissolving 20 µl of extracted ACC, 80 µl of OPT and 900 µl of 70% ethanol. The mixture was mixed well and was allowed to stand for 30 min to complete the reaction and 20 µl of the prepared working samples were injected through a micro-injector. ACC-OPA derivative was detected through an attached fluorescence spectrophotometer at 1.0 ml/min flow rate using methanol as solvent A and acetonitrile as solvent B over 20 min. The calibration curve was prepared using standard ACC (Sigma Aldrich) to find the endogenous content in samples.

### In-vitro ovule culturing

The ovules of *G. barbadense* were cultured on BT medium with some modifications. BT medium was prepared according to^22^ with pH 5. The medium was supplemented with 0.75µM Gibberellic acid (GA_3_) and 5 µM and Indole acetic acid (IAA). BT media were either supplemented with 10 µM ACC or ethylene inhibitors i.e. 30 µM silver nitrate (AgNO_3_) or 40 µM cobalt chloride (CoCl_2_)^23,38^.

The flowers along with their anthocaulus (approximately 1 cm) were collected at the day of anthesis. Bracts, petals and stamens were removed carefully without hurting the pistil. The whole tissue was immediately immersed in 0.1% (w/v) of mercuric chloride (HgCl_2_) for 15 min with continuous shaking. Afterwards, the tissue was rinsed 4–5 times with autoclaved distilled water under standard aseptic conditions. The shuck of the ovary was removed carefully to avoid damage to the ovules. Ovules were then placed gently on the surface of the medium within each culture plate having 5 ml of nutrient medium. The floating ovules were incubated at 30 ± 1 °C in dark without shaking.

The cultured ovules (21 DPA) were immersed in 75% (v/v) ethanol for 20 min. They were then placed on a clean glass slide and kept under running water to make their one side flow. The lengths of the fibers were then measured with the help of a ruler.

## Data Availability

All data generated or analysed during this study are included in this published article.
